# The influence of WeChat education and care program on anxiety, depression, insomnia, and general state of health in parents of pediatric acute lymphoblastic leukemia patients

**DOI:** 10.1007/s00432-024-05646-0

**Published:** 2024-03-19

**Authors:** Hui Duan, Li Wang, Hui Li, Zhongyu Wang, Shuili Jiao, Yanli Liu, Huihui Li, Jie Chen, Qiang Feng

**Affiliations:** 1 Department of Pediatrics, Affiliated Hospital of Hebei Engineering University, No. 81 Congtai Road, Handan, 056002 China; 2 Department of Intensive Care Unit, Hebei Engineering University Affiliated Hospital, Handan, 056000 China; 3 Department of Oncology 4, Handan Central Hospital, Handan, 056002 China; 4 Department of Pediatrics Ward 2, Handan Central Hospital, Handan, 056002 China; 5 Department of Neonatology Ward 1, Handan Central Hospital, Handan, 056002 China; 6 Department of Nephrology 2, Handan Central Hospital, Handan, 056002 China; 7 Department of Cardiology 4, Handan Central Hospital, Handan, 056002 China

**Keywords:** Parents of pediatric acute lymphoblastic leukemia patients, WeChat education and care program, Anxiety and depression, Insomnia, General state of health

## Abstract

**Purpose:**

WeChat-based education and care program serves as a promising nursing method for relieving mental stress in parents of pediatric patients. This study purposed to explore the influence of the WeChat education and care program (WECP) on mental health, insomnia, and general state of health in parents of pediatric acute lymphoblastic leukemia (ALL) patients.

**Methods:**

Totally, 146 parents of 73 primary pediatric ALL patients were randomized into the WECP group (74 parents of 37 patients) and standard care (SC) group (72 parents of 36 patients) to receive a 6-month corresponding intervention. Self-rating anxiety scale (SAS), self-rating depression scale (SDS), Athens insomnia scale (AIS), and 12-item general health questionnaire (GHQ-12) were assessed in parents of patients.

**Results:**

SAS scores at the third month (M3) (*P* = 0.041) and M6 (*P* = 0.032) were reduced in WECP group versus SC group. SAS-defined anxiety rate at M6 (*P* = 0.035) was declined in WECP group versus SC group. SDS score at M6 was descended in WECP group versus SC group (*P* = 0.024). However, there was no discrepancy in SDS-defined depression rate at any time point between groups (all *P* > 0.05). AIS scores at M1 (*P* = 0.015) and M6 (*P* = 0.021), as well as GHQ-12 scores at M3 (*P* = 0.007) and M6 (*P* = 0.001) were decreased in WECP group versus SC group. By subgroup analyses, WECP exhibited good effects at M6 in mothers, but not in fathers.

**Conclusion:**

WECP is a feasible and efficacy intervention to improve mental stress and health status among parents of pediatric ALL patients, especially in mothers.

## Introduction

Acute lymphoblastic leukemia (ALL) is a hematological malignant tumor characterized by the overgrowth of immature lymphoid cells, which is the most frequent type of malignancy in children (Chang et al. 2021). Currently, the advancement of chemotherapy protocols and the development of supportive care have improved the prognosis of pediatric ALL patients, with a 5-year overall survival (OS) rate of 79.9%–90.4% (Hunger et al. 2012; Gunes et al. 2014; Ma H et al. 2014; Inaba and Pui 2021). However, the diagnosis of pediatric ALL imposes a serious burden on the physical and psychological health of patients’ parents (Mogensen et al. 2022; Ferraz et al. 2023). Evidence shows that a considerable proportion of parents of pediatric ALL patients suffer from mental distress, sleep problems, and reduced quality of life (Iqbal and Siddiqui 2002; Wang J et al. 2017; Rensen et al. 2022). Therefore, searching for effective interventions is a notable issue to relieve these symptoms in parents of pediatric ALL patients.

WeChat-based education and care program offers a promising nursing mode through the network platform, which addresses the spatial and temporal limitations of medical services (Xu et al. 2021; Wang Z et al. 2023). Notably, the application of WeChat-based education and care programs in the field of parents of child patients has been vastly explored (Yang et al. 2021; Wu et al. 2022). For example, one study illustrates that a WeChat-platform-based education and care program is effective in decreasing anxiety, depression, and post-traumatic stress disorder in parents of pediatric and adolescent patients with osteosarcoma (Wu et al. 2022). Another study suggests that WeChat follow-up management achieves the reduction of anxiety and depression, as well as the improvement of quality of life in parents of premature infants with patent ductus arteriosus (Yang et al. 2021). According to these studies, one hypothesis is proposed that nursing interventions based on WeChat may improve the physical and psychological health status of parents of pediatric ALL patients. However, no relevant studies have been conducted.

Therefore, the present study designed a WeChat education and care program (WECP), intending to investigate its effect on mental stress, insomnia, and the general state of health in parents of pediatric ALL patients.

## Methods

### Patients and parents

This randomized controlled study consecutively enrolled 146 parents of 73 primary pediatric ALL patients who were visited between May 2020 and June 2022. The inclusion criteria were: (1) patients were diagnosed as primary ALL; (2) patients were aged ≤ 18 years; (3) both parents were able to participate in this study (to remove the bias of parent's gender); and (4) both parents were skilled in using the WeChat (Tencent, China) application. The exclusion criteria were: (1) parents or patients who participated in other therapeutic interventions or clinical studies at the same time; (2) parents who had a documented mental illness; (3) mother who was pregnant or breastfeeding; and (4) parents who were reluctant to participate in this intervention method and had no commitment. This study was approved by the Ethics Committee. The written informed consent was signed by all parents. The written informed consent was required to be signed by the patient and his/her guardians if the patient was older than or equal to 8 years, and only by the guardians if the patient was younger than 8 years.

### Randomization of parents

The randomization of parents was based on the family (1 family had 1 patient and 2 parents). A block randomization method was used, and the block size was 4. The parents of each family were randomly divided into two groups in a ratio of 1:1. The parents obtained a randomized serial number (parents from the same family had the same serial number) which was placed in an opaque envelope with the grouping information. The above process was carried out by two uninformed nurses. Parents received their envelope after discharge. There were 72 parents of 36 patients in the standard care (SC) group and 74 parents of 37 patients in the WECP group.

### Treatment and interventions

This study did not intervene in the treatment of the patients. The treatment was chosen based on the patient's condition and willingness, as well as the doctor's recommendations, and the specific treatment regimen was mainly based on Chinese Childhood Leukemia Group (CCLG)-ALL-2008 or Chinese Childhood Cancer Group (CCCG)-ALL-2015.

After the patient was discharged, SC or WECP intervention was administered to their parents. Parents in the SC group were divided into different teams (8–10 parents per team) for SC, and each team was handled by 1–2 specialized nurses. The SC lasted a total of 6 months, and the main sections were as follows: (1) Pre-discharge education. Before discharge from the hospital, a 1-h offline education was provided by the nurses. The nurses distributed educational brochures to parents and provided them with psychological guidance. The nurses advised the parents to meditate, listen to music, jog, or read regularly to relieve their emotions. (2) Offline psychological and health support. The parents came to the hospital's rehabilitation training room every two weeks for 1-h face-to-face care. Nurses provided parents with relaxation exercises, including 20 min of massage, 20 min of meditation, and 20 min of free communication. (3) Telephone follow-up. The nurses conducted a weekly telephone follow-up with the parents via the phone numbers they left behind. The nurse would ask the parents about the current problems they were facing and provide them timely support.

Parents in the WECP group were divided into different teams (8–10 parents per team) for WECP, and each team was handled by 1–2 specialized nurses. The WECP lasted a total of 6 months. Parents in the WECP group received the same care as parents in the SC group, at the same time, parents in the WECP group received WeChat-based mental and physical health education. The main contents of WECP were as follows: (1) Followed the online public account. The nurses created a public account to upload the full content of the study materials, including educational articles and training videos (instructional videos on meditation and deep breathing). Parents who needed it could learn from the public account at any time. (2) Joined the online WeChat group. Through the WeChat group, parents could add the nurse's account, and if they encountered problems, they could communicate with the nurse one-on-one at any time. (3) Regular feedback on training. Each parent regularly uploaded videos of his/her meditation and deep breathing training to the assigned nurses, which was urged and checked by the nurses to ensure that the training was completed in a quality and quantity manner. (4) Parents communicated with each other in the WeChat group. Deep communication between parents started once a week in the WeChat group for at least an hour. Parents could share what they learned from the study, how they took care of their patients, or what troubles they had recently in their lives and work. The main purpose of the program was to let parents help each other. In addition to the professional education from doctors and nurses, parents could receive emotional support from other parents.

### Evaluations

Self-rating anxiety scale (SAS) score, SAS-defined anxiety, self-rating depression scale (SDS) score, SDS-defined depression, Athens insomnia scale (AIS) score, and 12-item general health questionnaire (GHQ-12) score evaluations were administered to parents before intervention (M0), and at the first (M1), third (M3), and sixth months (M6) after they received the intervention.

SAS scores and SAS-defined anxiety were used to measure the level of anxiety of the patients. The SAS score ranged from 25 to 100. Higher SAS scores indicated that the parent was more anxious. When the score was greater than or equal to 50, the parent was considered to be the anxiety. SDS scores and SDS-defined depression were used to measure the level of depression of the parents. The SDS score was ranged from 25 to 100. Higher SDS scores indicated that the parent was more depressed. When the score was greater than or equal to 50, the parent was considered to be the depression (Wang X et al. 2022). The AIS score measured the insomnia of the parents. The AIS score range was 0 to 24, with higher scores indicating more severe insomnia (Wang X et al. 2022). The GHQ-12 score measured the general state of health of the parents. The GHQ-12 score range was 0–12, with higher scores indicating a poorer state of health (Jin et al. 2020).

### Data analysis

The calculation of the minimum sample size was based on clinical experience. The SAS score at M6 in the SC group was predicted to be 45, and in the WECP group was predicted to be 50. The standard deviation (SD) was 10. The significance (α) level was 0.05 with a power of 80%. The minimum sample size was calculated to be 64. The study took into account that 10% of patients were lost to follow-up or disengaged, and the sample size was 72 per group. For data analysis, SPSS 24.0 (IBM Corp., New York, USA) software was used. Continuous variables were displayed by median (range), median and interquartile range (IQR), or mean ± SD. Categorical variables were demonstrated by numbers (percentages). Comparisons between the two groups were used with the Mann–Whitney U, Student’s t, Chi-square, and Fischer's exact tests. *P* < 0.05 were considered statistically different.

## Results

### Study flow

**Fig. 1:**
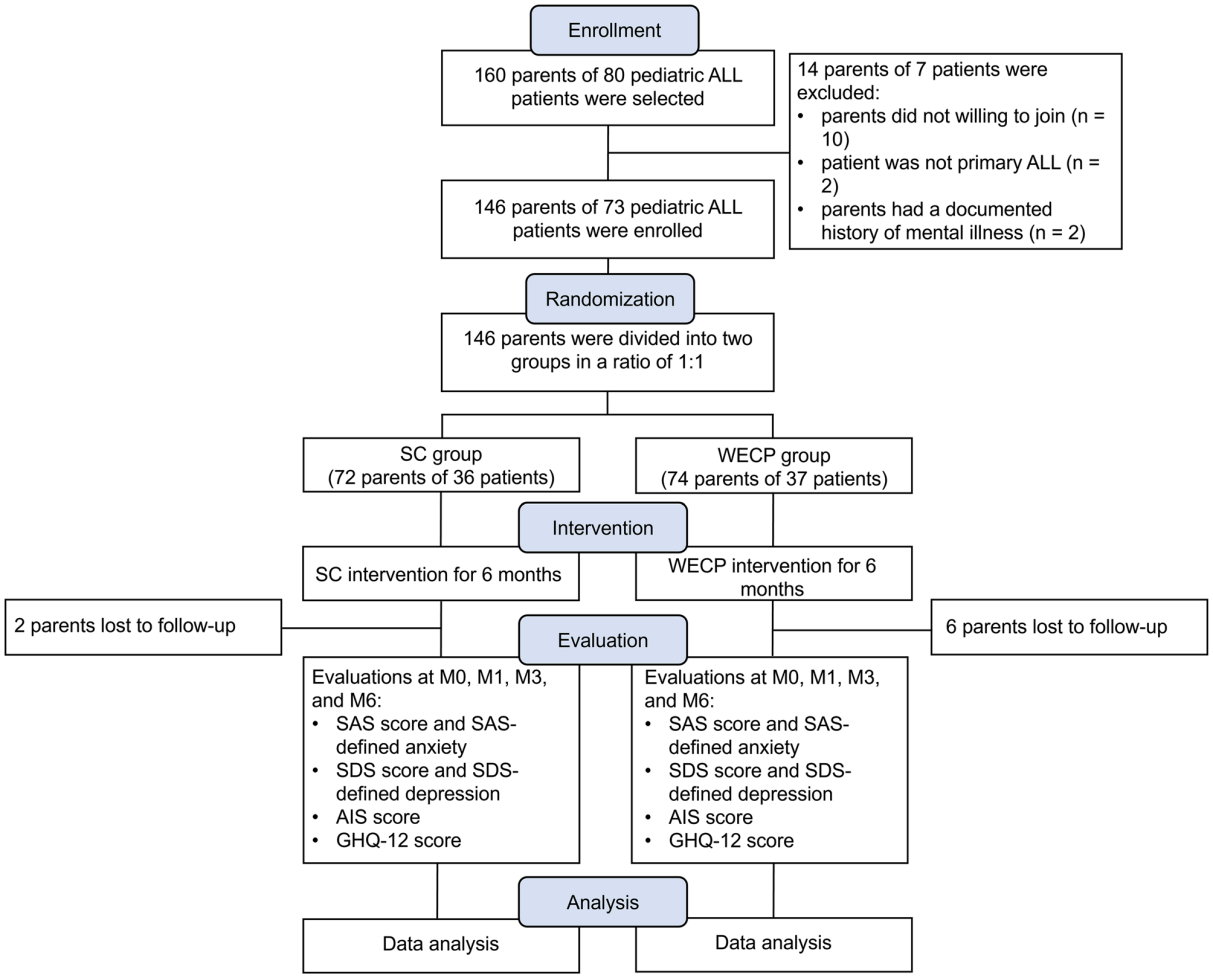
Study flow chart

In total, 160 parents of 80 pediatric ALL patients were selected, among which, 14 parents of 7 patients were excluded (including 10 parents who were not willing to join, 2 parents whose child was not primary ALL, and 2 parents of a patient due to a documented history of mental illness in one of the parents). Then, the eligible 146 parents of 73 pediatric ALL patients were enrolled and divided randomly into the WECP group (74 parents of 37 patients) and the SC group (72 parents of 36 patients) in a ratio of 1:1. In the WECP group, parents received WECP intervention for 6 months, and there were 6 parents who lost to follow-up. In the SC group, parents received SC intervention for 6 months, and there were 2 parents who lost to follow-up. SAS score and SAS-defined anxiety, SDS score and SDS-defined anxiety, AIS score, and GHQ-12 score were assessed at M0, M1, M3, and M6 (Fig. 1).

### Clinical traits between the two groups

The patients in the WECP group included 15 (40.5%) females and 22 (59.5%) males, and the median (range) age of patients was 4.0 (2.0–12.0) years. The patients in the SC group included 11 (30.6%) females and 25 (69.4%) males, and the median (range) age of patients was 4.0 (1.0–11.0) years. Regarding parents, the median (range) age of parents in the WECP group was 33.0 (26.0–44.0) years, and the median (range) age of parents in the SC group was 31.0 (25.0–44.0) years. There was no difference in all clinical traits of patients or parents between groups (all *P* > 0.05). More detailed clinical traits of patients and their parents are listed in Table 1.

**Table 1:** Characteristics of pediatric ALL patients and their parents

Characteristics	SC group	WECP group	*P* value
Pediatric ALL patients			
Age (years)^a^, median (range)	4.0 (1.0–11.0)	4.0 (2.0–12.0)	0.279
Gender^a^, *n* (%)			0.373
Female	11 (30.6)	15 (40.5)	
Male	25 (69.4)	22 (59.5)	
Height (cm)^a^, median (IQR)	107.5 (96.0–119.0)	109.0 (100.8–123.5)	0.251
Weight (kg)^a^, median (IQR)	18.4 (14.4–21.9)	19.2 (15.1–24.4)	0.354
Immunophenotype^a^, *n* (%)			0.801
T-ALL	6 (16.7)	7 (18.9)	
B-ALL	30 (83.3)	30 (81.1)	
FAB classification^a^, *n* (%)			0.402
L1	16 (44.5)	12 (32.4)	
L2	12 (33.3)	18 (48.7)	
L3	8 (22.2)	7 (18.9)	
WBC count > 50 X10^9^/L^a^, *n* (%)	8 (22.2)	5 (13.5)	0.331
Prednisone response at day 8^a^, *n* (%)			0.303
PPR	7 (19.4)	4 (10.8)	
PGR	29 (80.6)	33 (89.2)	
Bone marrow response at day 15^a^, *n* (%)			0.262
M1 (blasts < 5%)	27 (75.0)	32 (86.5)	
M2 (blasts 5 to < 25%)	8 (22.2)	3 (8.1)	
M3 (blasts ≥ 25%)	1 (2.8)	2 (5.4)	
Risk stratification^a^, *n* (%)			0.787
Low risk	14 (38.9)	15 (40.5)	
Intermediate risk	15 (41.7)	17 (45.9)	
High risk	7 (19.4)	5 (13.5)	
Parents			
Age (years)^b^, median (range)	31.0 (25.0–44.0)	33.0 (26.0–44.0)	0.057
Relation with the patients^b^, *n* (%)			1.000
Mother	36 (50.0)	37 (50.0)	
Father	36 (50.0)	37 (50.0)	
Level of education^b^, *n* (%)			0.682
Primary school	6 (8.3)	3 (4.1)	
Middle school	26 (36.1)	26 (35.1)	
High school	22 (30.6)	22 (29.7)	
Undergraduate or above	18 (25.0)	23 (31.1)	
Location^b^, *n* (%)			0.530
Rural	6 (8.3)	4 (5.4)	
Urban	66 (91.7)	70 (94.6)	
Annual household income (CNY)^c^, *n* (%)			0.810
< 30,000	1 (2.8)	1 (2.6)	
30,000–49,999	10 (27.8)	7 (18.9)	
50,000–99,999	17 (47.2)	17 (45.9)	
100,000–199,999	6 (16.7)	7 (18.9)	
≥ 200,000	2 (5.5)	5 (13.5)	

*ALL* acute lymphoblastic leukemia, *SC* standard care, *WECP* WeChat education and care program, *IQR* interquartile range, *T-ALL* T-cell acute lymphoblastic leukemia, *B-ALL* B-cell acute lymphoblastic leukemia, *FAB* French–American–British classification systems, *WBC* white blood cell, *PPR* prednisone poor response, *PGR* prednisone good response, *CNY* Chinese Yuan

^a^ 73 patients were analyzed

^b^146 parents were analyzed

^c^73 families were analyzed

### Anxiety and depression of parents in the two groups

SAS scores at M3 (44.8 ± 10.1 vs. 48.8 ± 12.3) (*P* = 0.041) and M6 (43.3 ± 9.3 vs. 47.3 ± 12.1) (*P* = 0.032) were decreased in the WECP group in comparison with the SC group. The SAS-defined anxiety rate at M3 tended to be lower in the WECP group in comparison with the SC group (30.9% vs. 47.1%) (*P* = 0.050). Notably, the SAS-defined anxiety rate at M6 was declined in the WECP group versus the SC group (29.2% vs. 47.1%) (*P* = 0.035) (Table 2).

**Table 2:** Comparison of outcomes for parents between SC and WECP groups

Outcomes	SC group	WECP group	*P* value
SAS score, mean ± SD			
M0	52.1 ± 13.1	51.9 ± 10.2	0.905
M1	50.3 ± 12.5	47.5 ± 10.5	0.151
M3	48.8 ± 12.3	44.8 ± 10.1	0.041
M6	47.3 ± 12.1	43.3 ± 9.3	0.032
SAS-defined anxiety, *n* (%)			
M0	40 (55.6)	40 (54.1)	0.855
M1	36 (50.0)	30 (42.3)	0.353
M3	33 (47.1)	21 (30.9)	0.050
M6	32 (47.1)	19 (29.2)	0.035
SDS score, mean ± SD			
M0	49.5 ± 11.5	48.5 ± 11.1	0.609
M1	47.8 ± 10.9	46.6 ± 9.3	0.459
M3	47.1 ± 9.8	44.1 ± 9.7	0.072
M6	46.5 ± 10.0	42.7 ± 9.1	0.024
SDS-defined depression, *n* (%)			
M0	31 (43.1)	31 (41.9)	0.887
M1	30 (41.7)	26 (36.6)	0.536
M3	28 (40.0)	20 (29.4)	0.192
M6	26 (38.2)	17 (26.2)	0.136

*SC* standard care, *WECP* WeChat education and care program, *SAS* self-rating anxiety scale, *SD* standard deviation, *SDS* self-rating depression scale, *M0* before intervention, *M1* first month after intervention, *M3* third month after intervention, *M6* sixth month after intervention

SDS score at M6 was descended in the WECP group compared to the SC group (42.7 ± 9.1 vs. 46.5 ± 10.0) (*P* = 0.024). While no discrepancy was found in the SDS-defined depression rate at any time point between the two groups (all *P* > 0.05) (Table 2).

### Insomnia and general state of health of parents in the two groups

**Fig. 2:**
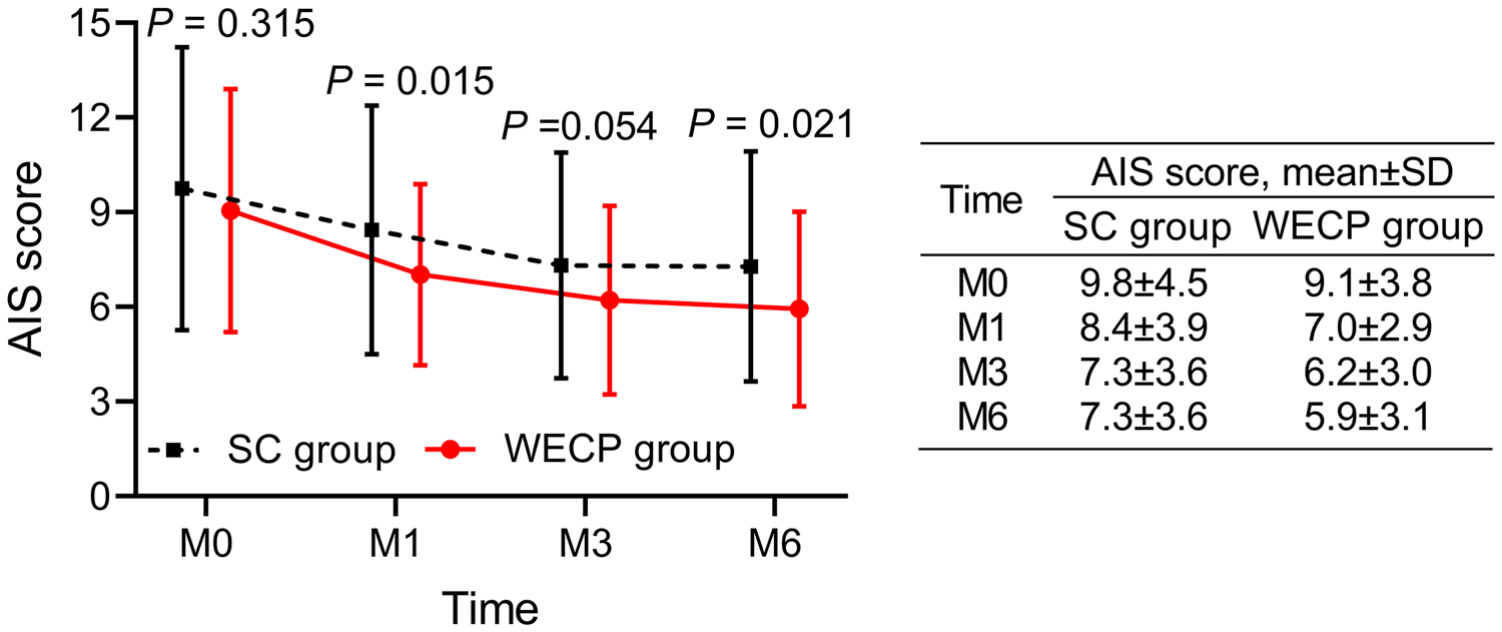
Comparison of AIS score of parents between groups

**Fig. 3:**
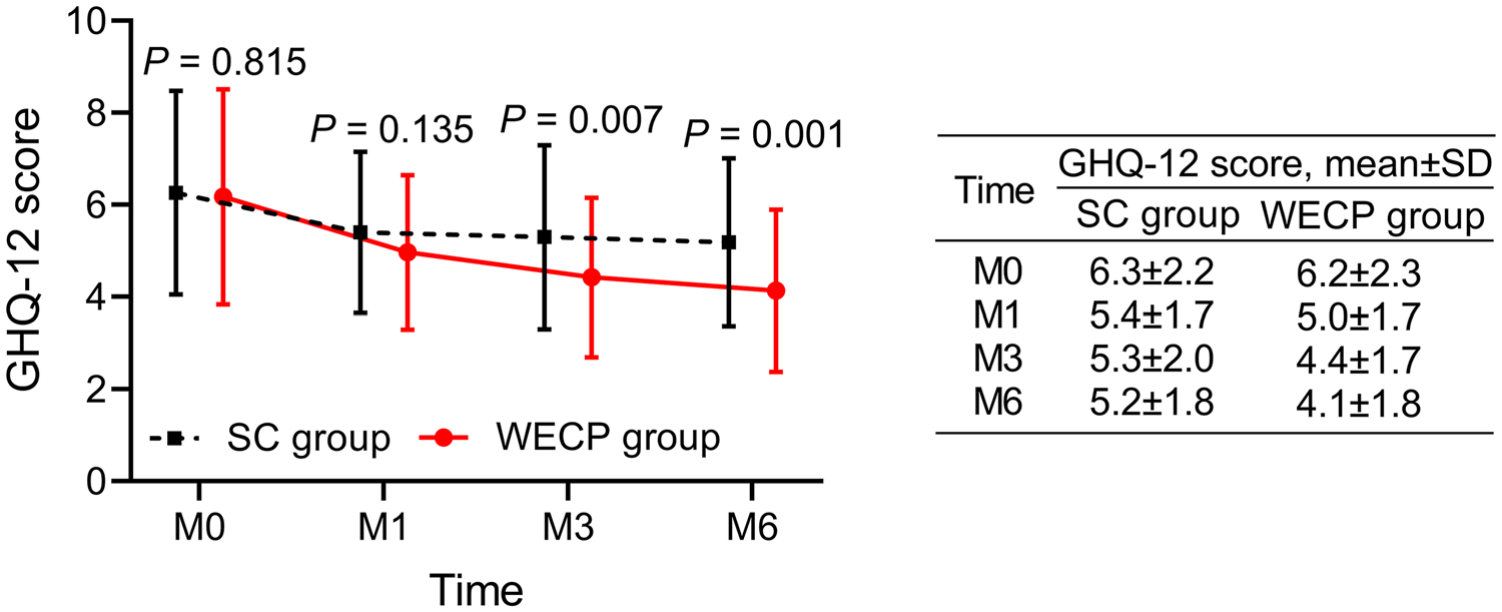
Comparison of GHQ-12 score of parents between groups

In terms of insomnia of parents, the AIS scores at M1 (7.0 ± 2.9 vs. 8.4 ± 3.9) (*P* = 0.015) and M6 (5.9 ± 3.1 vs. 7.3 ± 3.6) (*P* = 0.021) were decreased in the WECP group when compared to the SC group (Fig. 2). Regarding the general state of health of parents, the GHQ-12 scores at M3 (4.4 ± 1.7 vs. 5.3 ± 2.0) (*P* = 0.007) and M6 (4.1 ± 1.8 vs. 5.2 ± 1.8) (*P* = 0.001) were reduced in the WECP group in comparison with the SC group (Fig. 3).

### Subgroup analyses of the outcomes of parents at M6

The subgroup analyses were conducted based on the relationship between parents and pediatric ALL patients. In mothers, SAS score (41.6 ± 7.9 vs. 47.7 ± 13.1) (*P* = 0.026), the SAS-defined anxiety rate (15.6% vs. 50.0%) (*P* = 0.003), SDS score (42.0 ± 9.9 vs. 48.4 ± 8.8) (*P* = 0.008), and GHQ-12 score (4.1 ± 1.6 vs. 5.4 ± 1.8) (*P* = 0.004) at M6 were reduced in the WECP group in comparison with the SC group. In fathers, there was no difference in anxiety, depression, insomnia, or general state of health between the two groups (all* P* > 0.05) (Table 3).

**Table 3:** Subgroup analysis of the outcomes at M6 for parents based on the relationship with pediatric ALL patients

Outcomes	SC group	WECP group	*P* value
Mother			
SAS score, mean ± SD	47.7 ± 13.1	41.6 ± 7.9	0.026
SAS-defined anxiety, *n* (%)	17 (50.0)	5 (15.6)	0.003
SDS score, mean ± SD	48.4 ± 8.8	42.0 ± 9.9	0.008
SDS-defined depression, *n* (%)	16 (47.1)	9 (28.1)	0.113
AIS score, mean ± SD	7.5 ± 3.9	6.2 ± 3.5	0.161
GHQ-12 score, mean ± SD	5.4 ± 1.8	4.1 ± 1.6	0.004
Father			
SAS score, mean ± SD	47.0 ± 11.2	44.9 ± 10.4	0.436
SAS-defined anxiety, *n* (%)	15 (44.1)	14 (42.4)	0.889
SDS score, mean ± SD	44.6 ± 10.8	43.5 ± 8.2	0.614
SDS-defined depression, *n* (%)	10 (29.4)	8 (24.2)	0.633
AIS score, mean ± SD	7.1 ± 3.4	5.7 ± 2.7	0.061
GHQ-12 score, mean ± SD	5.0 ± 1.8	4.2 ± 1.9	0.074

*M6* sixth month after intervention, *ALL* acute lymphoblastic leukemia, *SC* standard care, *WECP* WeChat education and care program, *SAS* self-rating anxiety scale, *SD* standard deviation, *SDS* self-rating depression scale, *AIS* Athens insomnia scale, *GHQ-12* 12-item general health questionnaire

## Discussion

Parents of children with malignancies often inevitably develop a series of psychological problems (Agbayani et al. 2022). One previous study shows that anxiety and depression rates among parents of pediatric ALL patients are 45.4 and 57.7%, respectively (Wang et al. 2017). Another research illustrates that depression is observed among 56.7% of parents of pediatric ALL patients (Iqbal and Siddiqui 2002). In our study, baseline SAS/SDS-defined anxiety and depression rates in parents of pediatric ALL patients were 54.1–55.6% and 41.9–43.1%, respectively. The baseline anxiety and depression rates in our study differed slightly from previous studies (Iqbal and Siddiqui 2002; Wang et al. 2017), which was possible because: (1) All patients in the previous study have high school education or above (Wang J et al. 2017), while a proportion of 44.4% parents in our study had less than a high school education. Thus, the differences in the included population might lead to different results. (2) The previous study assesses depression through the Mini Mental State Examination (MMSE) and Structured Clinical Interview according to the Diagnostic and Statistical Manual of Mental Disorders-fourth version (SCID-IV) (Iqbal and Siddiqui 2002), while our study evaluated depression by SDS. Therefore, the different results in depression rates might be due to the differences in assessment scales. In any case, searching for effective nursing interventions is a key issue in relieving the anxiety and depression of parents of pediatric ALL patients (Wang et al. 2018).

As the main communication tool of contemporary people, WeChat has broken the limitations of time and space required by ordinary nursing, which provides a more convenient nursing mode (Xu et al. 2021; Huang et al. 2023). In our study, WECP effectively achieved the reduction of SAS/SDS-defined anxiety and depression versus SC in parents of pediatric ALL patients. There were several possible explanations: (1) WECP strengthened psychological education for parents through the online public account, which enabled parents to understand their mental problems conveniently and take corresponding measures timely (Hu et al. 2021). (2) WECP provided a platform for parents to consult nurses one-on-one when they encountered psychological issues, and the pressure might be relieved effectively under the guidance of nurses (Ma C et al. 2021; Wu et al. 2022). (3) WECP promoted communication among parents, which made parents share learning experiences, release negative emotions, and gain encouragement from each other (Luo et al. 2019). Thus, WECP improved the mental health of parents of pediatric ALL patients. In addition, our study also found that WECP relieved insomnia and improved the general state of health in parents of pediatric ALL patients. These findings were probably due to the fact that: (1) WECP provided instructional videos through the online public account, which encouraged parents to train without the constraints of time and space, thus increasing the training efficacy. Meanwhile, meditation and deep breathing training relieved the pressure on parents by regulating the autonomic nervous system, thereby reducing insomnia (Jerath et al. 2018). (2) In WECP, the nurses checked parent’s training regularly to ensure the training quality and quantity, which enhanced the parent’s self-care ability and physical status, thus improving the general state of health (Ma D et al. 2018; Luo et al. 2019). (3) Based on the results of our study, WECP alleviated the pressure and elevated the sleep quality of parents, which might contribute to the improvement of the general state of health (Schneiderman et al. 2005; Matsui et al. 2021).

In addition, subgroup analyses exhibited that the WECP yielded better outcomes versus SC in mothers of pediatric ALL patients, while there was no difference in outcomes in fathers between the two groups. These findings indicated that WECP might be more applicable to mothers of pediatric ALL patients versus fathers. This result might be attributed to the following points: (1) Compared with fathers, mothers might have a greater need for emotional venting and social support (Clarke et al. 2009; Wan Ghazali et al. 2023), and WECP satisfied their needs by building an online platform. Thus, WECP might be more effective for mothers of pediatric ALL patients. (2) Compared with men, women might be more willing to actively seek for help through social media when facing psychological or physical problems (Judd et al. 2008; Smail-Crevier et al. 2019). Therefore, WECP worked better among mothers of pediatric ALL patients. However, more studies were needed for verification.

Notably, our study excluded parents who had a documented mental illness and mothers who were pregnant or breastfeeding. The reasons were as follows: (1) Parents who had a documented mental illness might not be suitable to participate in therapeutic interventions or clinical studies; meanwhile, their mental illness might bias the results to a certain extent. Moreover, previous studies also excluded parents who have a documented mental illness (Ma et al. 2021; Xu et al. 2021; Wu et al. 2022). (2) Clinical studies usually exclude mothers who are pregnant or breastfeeding due to concerns about possible harm to these mothers and their infants (Ma et al. 2021; Kang and Li 2022; Leung et al. 2023). Thus, our study also excluded mothers who were pregnant or breastfeeding.

Our study had some limitations: (1) Data show that parents of pediatric ALL patients may report mental stress, sleep problems, and reduced quality of life in the long term (Rensen et al. 2022), while the intervention and follow-up period of our study was only 6 months. Therefore, the long-term effect of WECP on parents of pediatric ALL patients was required to be investigated. (2) Parents of pediatric ALL patients were easily aware of their grouping during the intervention period, which might cause biased results. (3) The assessment scales were single in our study, and further studies with multiple assessment scales were needed to comprehensively evaluate physical and psychological symptoms in parents of pediatric ALL patients.

## Conclusion

In conclusion, WECP is helpful for parents of pediatric ALL patients to relieve anxiety, depression, and insomnia, as well as improve health status, whose effects are better for mothers.

## Data Availability

The data used to support the findings of this study are available from the corresponding author upon request.

## References

[CR1] Agbayani CJ, Tucker JA, Nelson EL et al (2022) Immunological and psychosocial functioning in parents of children with cancer. Support Care Cancer 30(4):3379–3388. 10.1007/s00520-021-06770-0 34994860 10.1007/s00520-021-06770-0 PMC9833860

[CR2] Chang JH, Poppe MM, Hua CH et al (2021) Acute lymphoblastic leukemia. Pediatr Blood Cancer 68(Suppl 2):e28371. 10.1002/pbc.28371 33818880 10.1002/pbc.28371

[CR3] Clarke NE, McCarthy MC, Downie P et al (2009) Gender differences in the psychosocial experience of parents of children with cancer: a review of the literature. Psychooncology 18(9):907–915. 10.1002/pon.1515 19319828 10.1002/pon.1515

[CR4] Ferraz A, Santos M, Pereira MG (2023) Parental distress in childhood acute lymphoblastic leukemia: a systematic review of the literature. J Fam Psychol. 10.1037/fam0001113 37289501 10.1037/fam0001113

[CR5] Gunes AM, Oren H, Baytan B et al (2014) The long-term results of childhood acute lymphoblastic leukemia at two centers from Turkey: 15 years of experience with the ALL-BFM 95 protocol. Ann Hematol 93(10):1677–1684. 10.1007/s00277-014-2106-0 24863691 10.1007/s00277-014-2106-0

[CR6] Hu J, Cai Z, Ma X (2021) Effects of WeChat-based psychological interventions on the mental health of patients with suspected new coronavirus pneumonia: a pilot study. Jpn J Nurs Sci 18(4):e12429. 10.1111/jjns.12429 33999501 10.1111/jjns.12429 PMC8420323

[CR7] Huang X, Kang Y, Wang M et al (2023) WeChat-based remote follow-up management reduces the burden of home care and anxiety on parents of children with refractory epilepsy: a randomized controlled study. Medicine (baltimore) 102(25):e34070. 10.1097/MD.0000000000034070 37352033 10.1097/MD.0000000000034070 PMC10289595

[CR8] Hunger SP, Lu X, Devidas M et al (2012) Improved survival for children and adolescents with acute lymphoblastic leukemia between 1990 and 2005: a report from the children’s oncology group. J Clin Oncol 30(14):1663–1669. 10.1200/JCO.2011.37.8018 22412151 10.1200/JCO.2011.37.8018 PMC3383113

[CR9] Inaba H, Pui CH (2021) Advances in the diagnosis and treatment of pediatric acute lymphoblastic leukemia. J Clin Med. 10.3390/jcm10091926 33946897 10.3390/jcm10091926 PMC8124693

[CR10] Iqbal A, Siddiqui KS (2002) Depression among parents of children with acute lymphoblastic leukemia. J Ayub Med Coll Abbottabad 14(2):6–9 12238347

[CR11] Jerath R, Beveridge C, Barnes VA (2018) Self-regulation of breathing as an adjunctive treatment of insomnia. Front Psychiatry 9:780. 10.3389/fpsyt.2018.00780 30761030 10.3389/fpsyt.2018.00780 PMC6361823

[CR12] Jin Y, Zhang YS, Zhang Q et al (2020) Prevalence and socio-demographic correlates of poor mental health among older adults in agricultural areas of China. Front Psychiatry 11:549148. 10.3389/fpsyt.2020.549148 33250790 10.3389/fpsyt.2020.549148 PMC7674548

[CR13] Judd F, Komiti A, Jackson H (2008) How does being female assist help-seeking for mental health problems? Aust N Z J Psychiatry 42(1):24–29. 10.1080/00048670701732681 18058440 10.1080/00048670701732681

[CR14] Kang K, Li S (2022) A WeChat-based caregiver education program improves satisfaction of stroke patients and caregivers, also alleviates poststroke cognitive impairment and depression: a randomized, controlled study. Medicine (baltimore) 101(27):e29603. 10.1097/MD.0000000000029603 35801782 10.1097/MD.0000000000029603 PMC9259181

[CR15] Leung F, Miljanic S, Fernandes V et al (2023) Eligibility and enrollment of pregnant and breastfeeding women in psychiatry randomized controlled trials. Arch Womens Ment Health 26(3):353–359. 10.1007/s00737-023-01319-y 37106141 10.1007/s00737-023-01319-y PMC10139916

[CR16] Luo J, Dong X, Hu J (2019) Effect of nursing intervention via a chatting tool on the rehabilitation of patients after Total hip Arthroplasty. J Orthop Surg Res 14(1):417. 10.1186/s13018-019-1483-4 31818330 10.1186/s13018-019-1483-4 PMC6902538

[CR17] Ma H, Sun H, Sun X (2014) Survival improvement by decade of patients aged 0–14 years with acute lymphoblastic leukemia: a SEER analysis. Sci Rep 4:4227. 10.1038/srep04227 24572378 10.1038/srep04227 PMC3936227

[CR18] Ma D, Cheng K, Ding P et al (2018) Self-management of peripherally inserted central catheters after patient discharge via the WeChat smartphone application: a systematic review and meta-analysis. PLoS&nbsp;ONE 13(8):e0202326. 10.1371/journal.pone.0202326 10.1371/journal.pone.0202326 PMC6112638 30153253

[CR19] Ma C, Wang B, Zhao X et al (2021) WeChat-based education and rehabilitation program in unprotected left main coronary artery disease patients after coronary artery bypass grafting: an effective approach in reducing anxiety, depression, loss to follow-up, and improving quality of life. Braz J Med Biol Res 54(4):e10370. 10.1590/1414-431X202010370 33624731 10.1590/1414-431X202010370 PMC7894386

[CR20] Matsui K, Yoshiike T, Nagao K et al (2021) Association of subjective quality and quantity of sleep with quality of life among a general population. Int J Environ Res Public Health. 10.3390/ijerph182312835 34886562 10.3390/ijerph182312835 PMC8657737

[CR21] Mogensen N, Saaranen E, Olsson E et al (2022) Quality of life in mothers and fathers of children treated for acute lymphoblastic leukaemia in Sweden Finland Denmark. Br J Haematol 198(6):1032–1040. 10.1111/bjh.18350 35852790 10.1111/bjh.18350 PMC9543076

[CR22] Rensen N, Steur L, Grootenhuis M et al (2022) Parental sleep, distress, and quality of life in childhood acute lymphoblastic leukemia: a longitudinal report from diagnosis up to three years later. Cancers (Basel). 10.3390/cancers14112779 35681759 10.3390/cancers14112779 PMC9179657

[CR23] Schneiderman N, Ironson G, Siegel SD (2005) Stress and health: psychological, behavioral, and biological determinants. Annu Rev Clin Psychol 1:607–628. 10.1146/annurev.clinpsy.1.102803.144141 17716101 10.1146/annurev.clinpsy.1.102803.144141 PMC2568977

[CR24] Smail-Crevier R, Powers G, Noel C et al (2019) Health-related internet usage and design feature preference for E-Mental health programs among men and women. J Med Internet Res 21(3):e11224. 10.2196/11224 30882361 10.2196/11224 PMC6441854

[CR25] Wan Ghazali WS, Minhat HS, Mohd Zulkefli NA et al (2023) Systematic review on factors associated with depression among mothers of children with cancer. PLoS&nbsp;ONE 18(8):e0285366. 10.1371/journal.pone.0285366 10.1371/journal.pone.0285366 PMC10449221 37616287

[CR26] Wang J, Shen N, Zhang X et al (2017) Care burden and its predictive factors in parents of newly diagnosed children with acute lymphoblastic leukemia in academic hospitals in China. Support Care Cancer 25(12):3703–3713. 10.1007/s00520-017-3796-3 28714044 10.1007/s00520-017-3796-3

[CR27] Wang J, Howell D, Shen N et al (2018) mHealth supportive care intervention for parents of children with acute lymphoblastic leukemia: quasi-experimental pre- and postdesign study. JMIR Mhealth Uhealth 6(11):e195. 10.2196/mhealth.9981 30455166 10.2196/mhealth.9981 PMC6301810

[CR28] Wang X, Chen J, Liu YE et al (2022) The effect of acceptance and commitment therapy on psychological nursing of acute cerebral infarction with insomnia, anxiety, and depression. Comput Math Methods Med 2022:8538656. 10.1155/2022/8538656 35785139 10.1155/2022/8538656 PMC9242814

[CR29] Wang Z, Deng S, Lv H et al (2023) Effect of WeChat-based continuous care intervention on the somatic function, depression, anxiety, social function and cognitive function for cancer patients: Meta-analysis of 18 RCTs. Nurs Open 10(9):6045–6057. 10.1002/nop2.1916 37365734 10.1002/nop2.1916 PMC10416022

[CR30] Wu J, Meng J, Li H (2022) WeChat-platform-based education and care program as a candidate approach to relieve anxiety, depression, and post-traumatic stress disorder in parents of pediatric and adolescent patients with osteosarcoma. Front Psychol 13:913940. 10.3389/fpsyg.2022.913940 36092098 10.3389/fpsyg.2022.913940 PMC9453747

[CR31] Xu M, Yang X, Liu L et al (2021) Effect of the WeChat platform health management and refined continuous nursing model on life quality of patients with acute myocardial infarction after PCI. J Healthc Eng 2021:5034269. 10.1155/2021/5034269 34880976 10.1155/2021/5034269 PMC8648441

[CR32] Yang B, Liu JF, Xie WP et al (2021) The effects of WeChat follow-up management to improve the parents’ mental status and the quality of life of premature newborns with patent ductus arteriosus. J Cardiothorac Surg 16(1):235. 10.1186/s13019-021-01617-5 34419135 10.1186/s13019-021-01617-5 PMC8379576

